# Antibiofilm Substantivity of AgNPs in Clinical Oral Biofilms from Patients with Motor and Intellectual Disabilities: An In Vitro and Exploratory Study

**DOI:** 10.3390/ph19081299

**Published:** 2026-08-17

**Authors:** Carolina Holguín-Meraz, Rita Elizabeth Martínez-Martínez, Erasto Armando Zaragoza-Contreras, Rubén Abraham Domínguez-Pérez, Karla Lizette Tovar-Carrillo, Alejandro Donohue-Cornejo, Juan Carlos Cuevas-González, Simón Yobanny Reyes-López, Erika de Lourdes Silva-Benítez, María Verónica Cuevas-González, León Francisco Espinosa-Cristóbal

**Affiliations:** 1 Master Program in Dental Sciences, Stomatology Department, Institute of Biomedical Sciences, Autonomous University of Juarez City (UACJ), Envolvente del PRONAF and Estocolmo s/n, Ciudad Juárez 32310, Chihuahua, Mexico; al220733@alumnos.uacj.mx (C.H.-M.); karla.tovar@uacj.mx (K.L.T.-C.); adonohue@uacj.mx (A.D.-C.); juan.cuevas@uacj.mx (J.C.C.-G.); maria.cuevas@uacj.mx (M.V.C.-G.); 2 Master Program in Advanced Dentistry, Faculty of Dentistry, Autonomous University of San Luis Potosí, Manuel Nava Avenue, University Campus, San Luis Potosí 78290, San Luis Potosí, Mexico; ritae_martinez@hotmail.com; 3 Department of Engineering and Materials Chemistry, Centro de Investigación en Materiales Avanzados, S. C., Miguel de Cervantes No.120, Chihuahua 31136, Chihuahua, Mexico; armando.zaragoza@cimav.edu.mx; 4 Laboratory of Multidisciplinary Dental Research, Center for Advanced Biomedical Research, Faculty of Medicine, Autonomous University of Queretaro, Chichimequillas and Universidad Avenues, Ejido Bolaños, Santiago de Querétaro 76140, Querétaro, Mexico; dominguez.ra@uaq.mx; 5 Institute of Biomedical Sciences, Autonomous University of Juarez City (UACJ), Envolvente del PRONAF and Estocolmo s/n, Ciudad Juárez 32310, Chihuahua, Mexico; simon.lopez@uacj.mx; 6 Faculty of Odontology, Autonomous University of Sinaloa, Josefa Ortiz de Domínguez Street, Universitary Campus, Universitaria, Culiacán 80010, Sinaloa, Mexico; erikasilva@uas.edu.mx

**Keywords:** anti-infective agents, biofilms, dental caries, dental disinfectants, Down syndrome, intellectual disabilities, metal nanoparticles, periodontal diseases, persons with disabilities, silver

## Abstract

**Background**: Motor and intellectual disabilities (MIDs) belong to a restricted and vulnerable social group with health restrictions for maintaining and preserving adequate oral health. Silver nanoparticles (AgNPs) have appeared as an encouraging therapy due to their excellent antimicrobial effects; however, scientific information on the antimicrobial effectiveness of AgNPs against bacteria in patients with MIDs remains limited. This study aimed to evaluate the antimicrobial substantivity of AgNPs in oral bacteria from patients with and without MIDs. **Methods**: Fifty-four oral biofilm samples were obtained from 26 participants with MIDs and 28 participants without MIDs. The microbial inhibition activity and the antimicrobial substantivity of AgNPs were determined. The antibiofilm activity of the AgNPs was explored using scanning electron microscopy (SEM). **Results**: The AgNPs displayed uniform size distributions (9.8 ± 1.7 nm) and electrical charges in surface particles that limit particle clustering (−39.8 ± 3.3 mV). The AgNPs were more effective than chlorhexidine (CHX) in eliminating bacteria from oral biofilms derived from both MID and non-MID patients (*p* < 0.05); therefore, the antimicrobial substantivity of the AgNPs was statistically better than deionized water but lower than CHX at specific exposure times (*p* < 0.05). The antimicrobial effectiveness of AgNPs at even low concentrations was associated with the type of oral biofilm, the type of antimicrobial solution, and, in some cases, gender. SEM images indicate a clear alteration in oral biofilm structure when AgNPs were applied. **Conclusions**: AgNPs exhibited significant potential to limit oral bacterial proliferation from microbial biofilms while contributing to the maintenance of oral condition in individuals with MIDs.

## 1. Introduction

Motor and intellectual disabilities (MIDs) are mental and physical conditions that limit the daily life of patients, including by contributing to poorer health. According to the United Nations, nearly 1.3 billion individuals (15% of the world) have some type of incapacity. Unfortunately, 100 million of these individuals are children, increasing the risk of limited access to health care [1]. The World Health Organization (WHO) recognizes that people with disabilities are disproportionately affected by a range of health conditions, including depression, asthma, diabetes, stroke, obesity, and poor oral health, largely due to health inequities. Those health inequalities directly affect the quality of life of persons with disabilities, who continue to face stigma, discrimination, economic hardship, exclusion from educational and occupational opportunities, and other obstacles [2]. The Pan American Health Organization (PAHO) has reported that individuals, particularly those with cerebral palsy, Down syndrome, and depression, face environmental factors related to negative attitudes, limited transportation, and buildings with inaccessible routes, as well as limited social, medical, and oral supports, limiting their complete and effective participation in our society [3]. In Mexico, the National Institute of Statistics and Geography (INEGI, 2020) has reported that more than 6 million people (4.9%) have some type of disability, which affects their regular daily life [4].

It is very well known that people with MIDs suffer from health problems, including oral alterations, due to involuntary and non-controlled movements, or even low cognitive abilities [5,6]. Patients with MIDs have etiological factors that can predispose them to dental alterations (loss of teeth, bruxism, swallowing problems, and malocclusion, among others) and to oral diseases associated with bacterial aggregation (caries, gingivitis, periodontitis, and halitosis, among others) [6,7,8]. This bacterial aggregation is fundamental to the establishment and maturation of oral biofilms, which are highly organized multispecies microbial communities [9]. Oral biofilm, which is constituted by polysaccharides, proteins, lipids, and minerals, grows in a matrix of extracellular polysaccharides (EPS), with a high potential for adhesion on different surfaces, including teeth and dental materials [10,11], offering them protection, structure, strength, and resistance against the maturation, proliferation, and dissemination of bacterial biofilm in the oral cavity [12,13]. Dental caries represents one of the most frequently occurring oral diseases and is strongly associated with the presence of bacterial biofilms, affecting 70% of people worldwide; it is also the most common condition among patients with MIDs and poor oral health [8,14]. Secondly, patients with MIDs can also develop bacterial resistance to antibiotics due to particular systemic conditions and persistent infections (congenital heart disease, immunodeficiencies, chronic and metabolic diseases, respiratory diseases, and undesirable effects from medications, among others) in which antimicrobials are used for long-term therapy [15,16]. Studies have reported that these factors can interfere with the oral health of patients with MIDs, such as Down syndrome, craniosynostosis, and congenital progeria, facilitating bacterial growth and promoting the growth of more metabolically aggressive bacteria, leading to dental caries or other biofilm-related bacterial diseases, such as periodontitis [17,18,19]. The most common bacteria contained in biofilms connected with the presence and progression of dental caries are, mainly, *Streptococcus mutans* and *Streptococcus sobrinus* and, secondarily, *Streptococcus salivarius* and *Streptococcus sanguinis*, among others [20], while the most frequent species for periodontal disease in more severe stages are *Prevotella intermedia*, *Fusobacterium nucleatum*, *Aggregatibacter actinomycetemcomitans*, *Treponema denticola*, *Porphyromonas gingivalis*, and *Tannerella forsythia*, [21,22].

The dental therapies used to control the development and organization of oral biofilms have already been investigated thoroughly using mechanical and chemical agents [23,24,25]. The mechanical products are based on the physical disorganization of oral biofilms during the initial and maturation stages, essentially using toothbrushes, dental floss, toothpicks, and various micro-abrasive materials included in toothpastes. On the other hand, chemical solutions have been developed as a complementary function parallel to mechanical products to avoid, limit, or decelerate the formation of oral biofilms. The most common and effective solutions for daily oral health care include chemical agents based on fluorides (including in cooking salt [26], toothpastes [27], mouthrinses [23], varnishes [28], and water for consumption [29], among others [30]) and chlorhexidine (CHX, the gold-standard solution in dentistry and used typically in mouthwashes for controlling periodontal infections) [31]. In the case of CHX, the antimicrobial substantivity (extended residual activity) is a property that helps to maintain the antimicrobial effect, even when the antimicrobial treatment has been removed [32]. This is an excellent characteristic that acts through prolonged absorption and release of the antimicrobial agent, preventing bacterial growth and bacterial reorganization during long-term intervals [33]. Despite this, fluoride and chlorhexidine solutions have not significantly reduced the prevalence of oral diseases related to oral biofilms [15], nor have they demonstrated any adverse effects with short-term administration [34]. In this sense, it is crucial to examine new approaches that explore novel materials and suitable mechanisms to control oral biofilms in patients with MIDs.

Silver nanoparticles (AgNPs) are engineered metallic nanomaterials widely recognized for their remarkable antimicrobial efficacy in a wide variety of species, including viruses, fungi, and bacteria, presenting significant potential for pharmaceutical and biomedical applications, including the field of dentistry [35,36,37,38]. Various studies have reported that AgNPs have the potential to generate adequate oral health through their antimicrobial, anti-adherence, and antibiofilm activity for various specialties of dentistry, interfering with clinical biofilms taken from patients with oral infections [39,40,41], even in oral biofilms from patients with MIDs [22]. Nevertheless, these studies lack sufficient sample sizes of oral biofilms, have limited distributions across MID types, and do not involve substantivity assays. This work aimed to examine the antimicrobial substantivity of AgNPs in oral biofilms recovered from individuals with and without MIDs. The null hypothesis was that AgNPs do not show antimicrobial activity or substantivity in microorganisms found in oral biofilms taken from individuals with motor and intellectual limitations using an in vitro methodological design. These results will improve the current knowledge of the antibacterial behavior of AgNPs in oral biofilms, as well as facilitate the advancement of novel metallic-nanomaterial-based therapies for the safe and effective control of bacterial oral diseases in individuals with MIDs.

## 2. Results

### 2.1. Physical Characterization of AgNP

**Figure 1:**
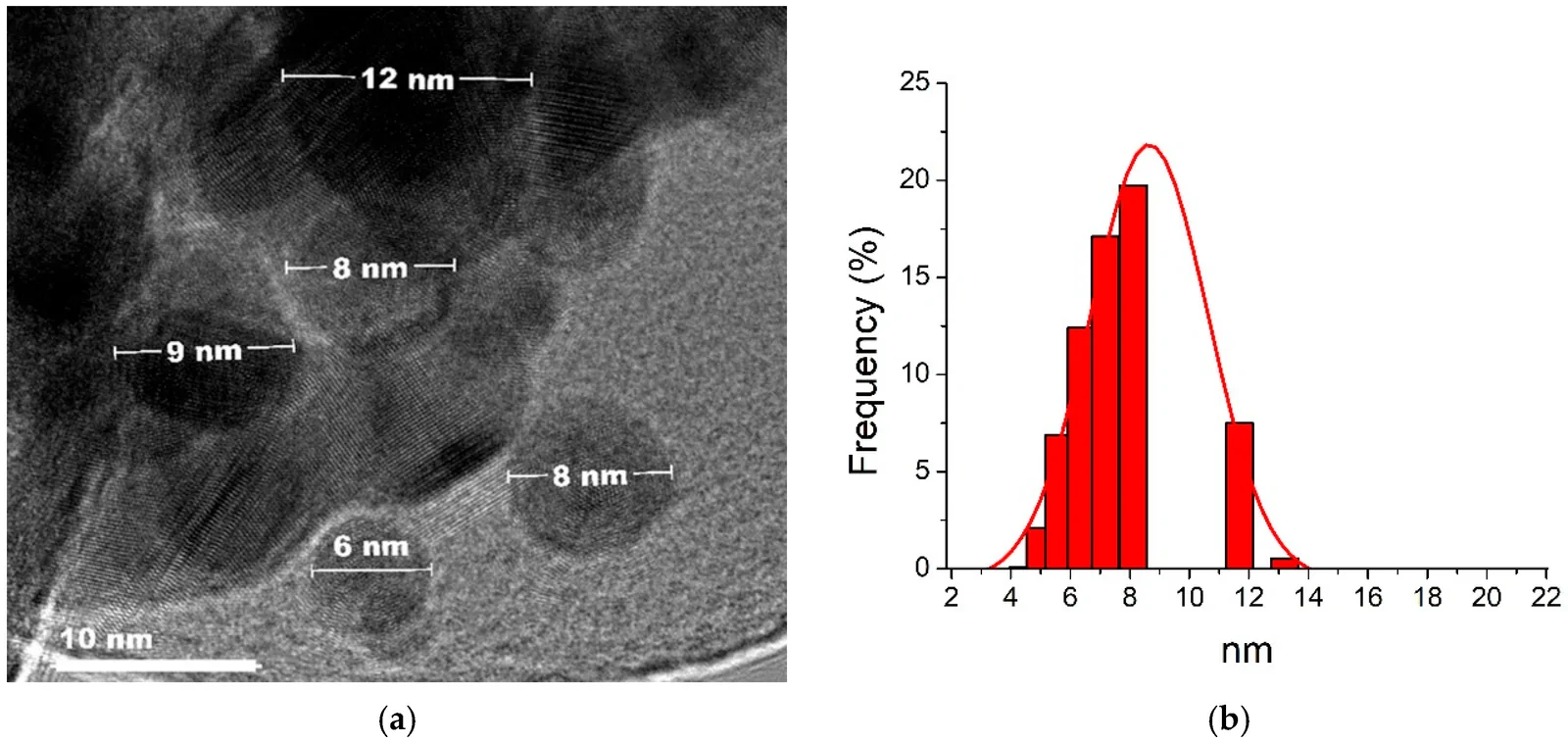
Physical characterization of the AgNPs: (**a**) TEM analysis, with the white bar indicating 10 nm; (**b**) DLS analysis (red bars indicate distribution of particles). TEM images were acquired using DigitalMicrograph^TM^ software, version 2.0 (Gatan Inc., Pleasanton, CA, USA).

The physical properties of the AgNPs are illustrated in Table 1 and Figure 1. Small and well-distributed particles with spherical shapes were obtained (9.8 ± 1.7 nm). DLS analysis indicated a single peak with a narrow basal line, suggesting a good particle distribution (Figure 1). The electrical charge of the particle surfaces was negative at high electrical intensities (−39.8 ± 3.3 mV), thereby promoting a low probability of particle agglomeration. These results denote that the AgNP solution exhibited a nanometric size with a uniform particle distribution, thereby limiting the risk of particle agglomeration.

### 2.2. Sociodemographic Distribution of Patients

The sociodemographic distributions of the patients with and without MIDs are presented in Table 2. Fifty-four oral biofilm samples were, generally, collected from children (9.1 ± 6.0 years). Autism was the most prevalent condition (*n* = 9 subjects), followed by cerebral palsy (*n* = 6 subjects), Down syndrome (*n* = 6 subjects), and, finally, craniosynostosis (*n* = 2 subjects), psychomotor retardation (*n* = 1 subject), Noonan syndrome (*n* = 1 subject), and congenital progeria (*n* = 1 subject). Participants with MIDs were significantly older (6.4–22 years) than those without disabilities (8.2 ± 0.3 years) (*p* < 0.05). Individuals with autism and craniosynostosis were slightly younger (6.4–7.0 years) than those with Noonan syndrome, cerebral palsy, and Down syndrome (9–12.1 years). Congenital progeria and psychomotor retardation represented the oldest group (19–22 years). Across all study groups, age was uniformly distributed between genders (*p* > 0.05). Although males predominated (55.6%) compared with females (44.4%), no statistically significant differences in sex distribution were observed between groups with and without MIDs (*p* > 0.05). Overall, these findings indicate a uniform gender distribution between groups, while age was generally higher among participants with MIDs.

### 2.3. Initial Bacterial Growth

**Figure 2:**
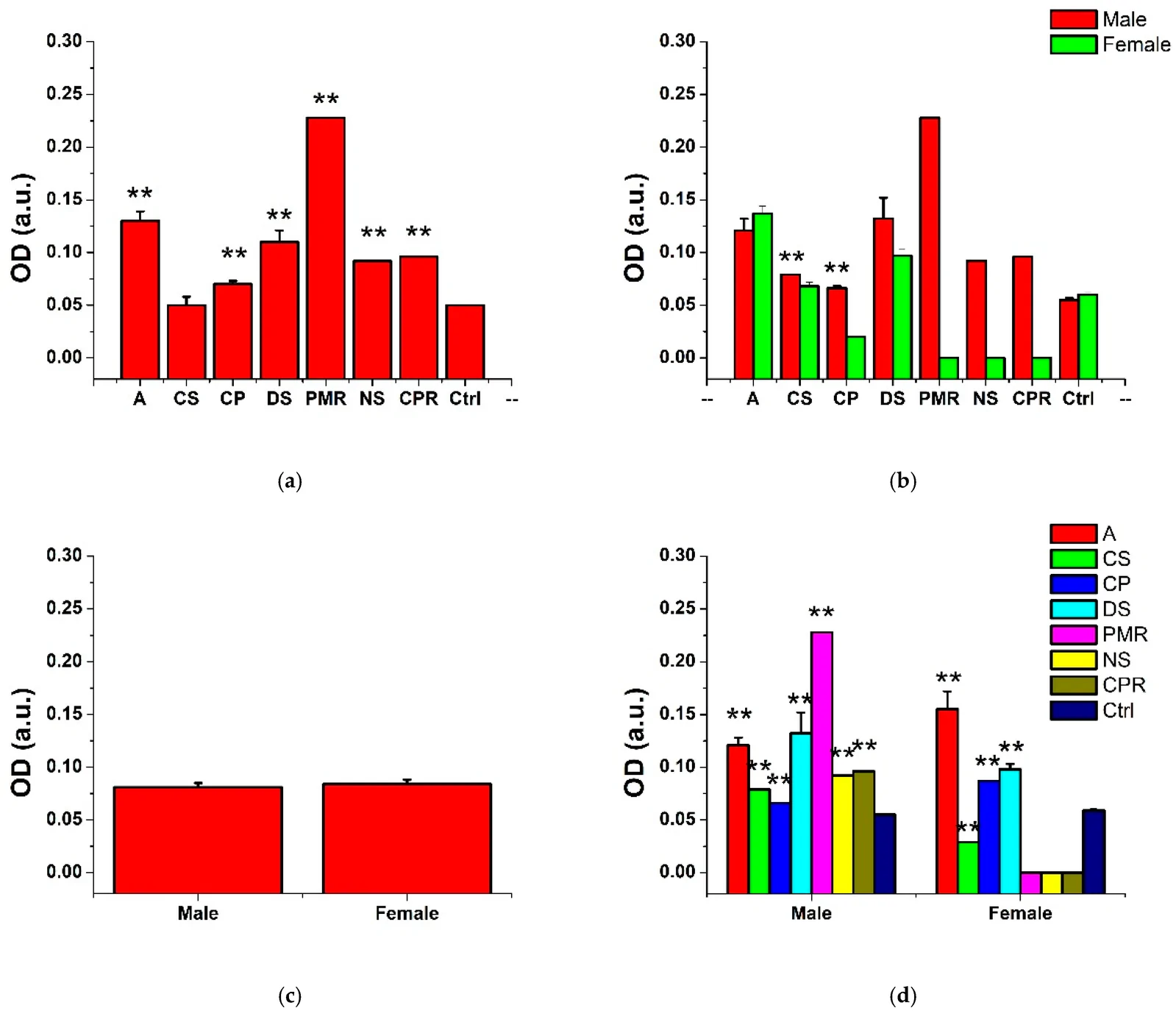
Initial microbial growth of biofilms obtained from individuals with motor and intellectual disabilities: (**a**) bacterial growth according to biofilm type; (**b**,**d**) bacterial growth stratified by biofilm type and sex; (**c**) bacterial growth according to sex. Asterisks denote statistically significant differences in comparison with the reference and female groups (** *p* < 0.01).

The initial microbial growth behavior of the oral biofilms linked with different MIDs is illustrated in Figure 2. Among the study groups, early microbial growth in these oral biofilms varied according to the bacterial cell proliferation. In general, bacterial proliferation was higher in the oral biofilms from patients with MIDs (0.07–0.22 a.u.) than in those from healthy controls (0.05 ± 0.0 a.u.) (*p* < 0.05), except for the CS group, where no statistically significant differences were reported (*p* > 0.05) (Figure 2a). Particularly, oral biofilms from individuals with PMR showed the highest microbial growth levels (0.22 ± 0.0 a.u.), followed by those from individuals with A (0.13 ± 0.0 a.u.), DS (0.11 ± 0.0 a.u.), CPR (0.09 ± 0.0 a.u.), NS (0.09 ± 0.0 a.u.), CP (0.07 ± 0.0 a.u.), and CS (0.05 ± 0.0 a.u.). Conversely, biofilms obtained from participants without disabilities exhibited the lowest microbial growth rates (0.05 ± 0.0 a.u.), with statistically significant differences also observed after stratification by sex (Figure 2a,d). With respect to gender, no significant differences in microbial proliferation rates were identified (*p* > 0.05) (Figure 2b,c). Particularly, male CS and CP patients demonstrated statistically more microbial cell growth (0.6–0.7 a.u.) than females (0.02–0.6 a.u.) (Figure 2b, *p* < 0.05). These results imply that initial bacterial growth is strongly influenced by both the type of oral biofilm and, in some cases, gender, with higher growth rates observed in biofilms from MID patients and male subjects.

### 2.4. Antimicrobial Activity of AgNP

**Figure 3:**
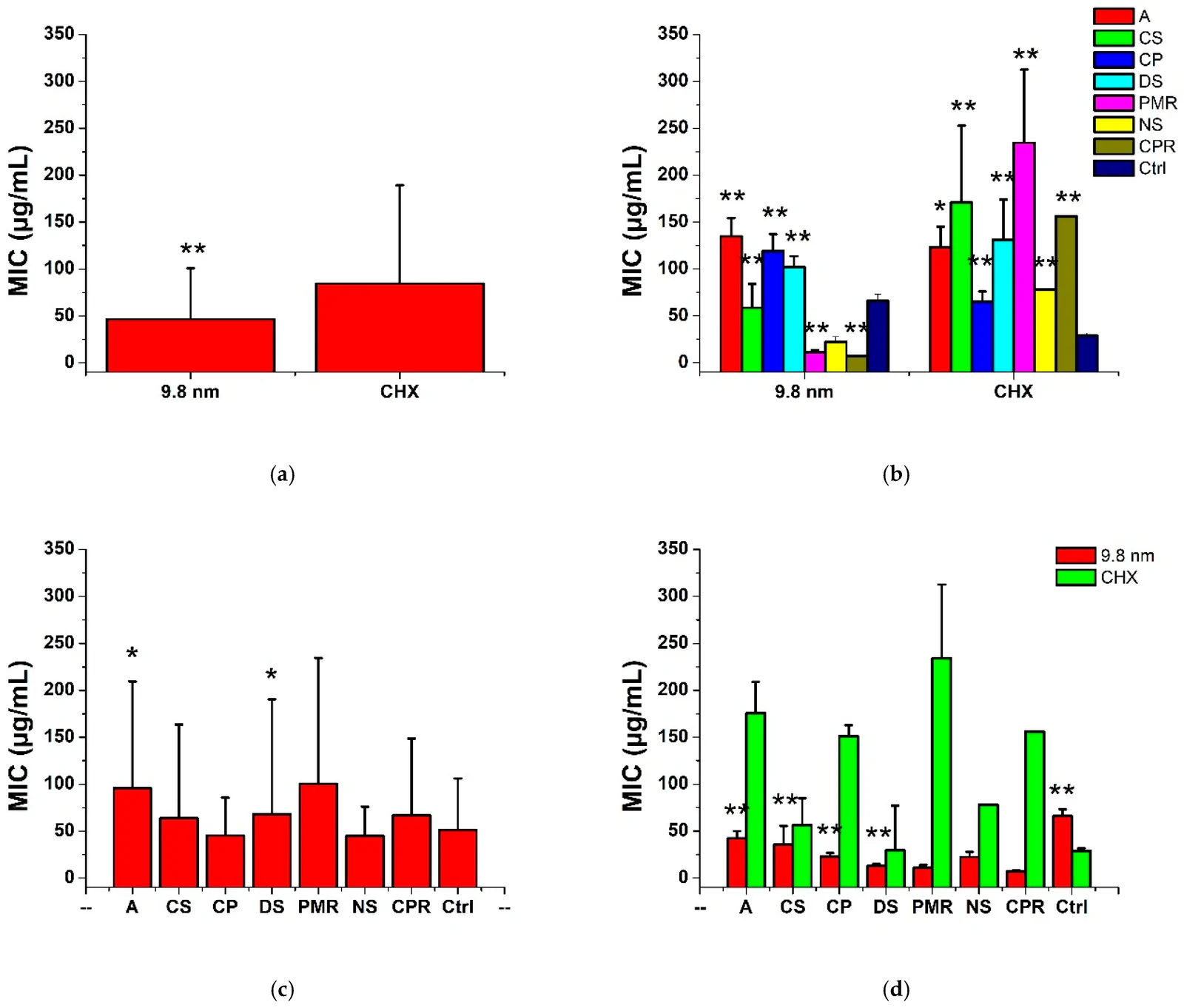
Antimicrobial effectiveness of AgNPs against oral biofilms from participants with MIDs: (**a**) antibacterial activity according to treatment group; (**b**) antibacterial activity based on treatment and oral biofilm type; (**c**) antimicrobial resistance according to oral biofilm type; (**d**) antibacterial resistance according to treatment and oral biofilm type. Asterisks denote statistically significant differences in comparison with the CHX and control (Ctrl) groups (* *p* < 0.05; ** *p* < 0.01).

The antibacterial impacts of AgNPs on oral biofilms from individuals with and without MIDs are shown in Table 3 and Figure 3. AgNPs demonstrated significantly lower MIC values (46.3 ± 54.6 µg/mL) compared with CHX (84.4 ± 104.7 µg/mL) (*p* = 0.000), indicating a stronger antimicrobial activity of AgNPs against oral biofilms for any disability status or gender (Figure 3a,d). In addition, some variations in MIC values were observed among the different types of oral biofilms; therefore, comparisons among the disability groups revealed no statistically significant differences (*p* = 0.129). Also, subjects with A (95.6 ± 113.9 µg/mL) and DS (68.0 ± 122.6 µg/mL) exhibited statistically higher resistance activity than non-disabled patients (51.5 ± 54.7 µg/mL) (*p* < 0.05, Figure 3c). In particular, all oral biofilms were statistically more resistant to CHX, while AgNPs had a low number of oral biofilms with antimicrobial resistance (four biofilms from seven samples) (Figure 3b). Additionally, biofilms from individuals with CP (45.2 ± 40.5 µg/mL) and NS (44.6 ± 31.3 µg/mL) showed the lowest MIC values, indicating a greater antimicrobial susceptibility to AgNPs, while biofilms from patients with PMR (100.4 ± 134.2 µg/mL) and A (95.6 ± 113.9 µg/mL) showed the most resistance behavior with no significant differences (*p* > 0.05). Finally, oral biofilms from healthy control subjects exhibited intermediate MIC values (51.5 ± 54.7 µg/mL).

**Figure 4:**
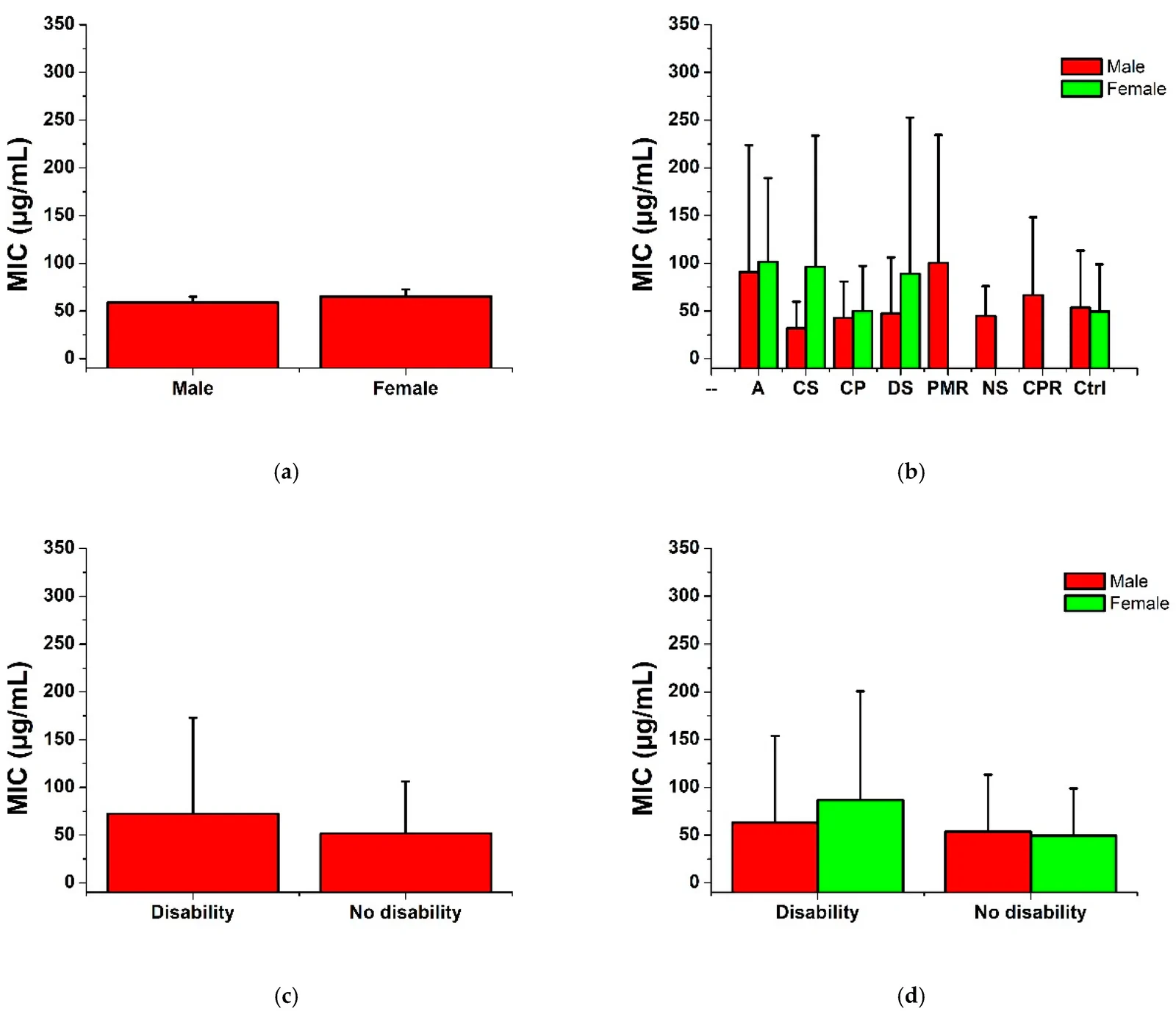
Assessment of AgNP-mediated antimicrobial activity in oral biofilms from participants with motor and intellectual incapacities stratified by sex: (**a**) general antibacterial activity according to gender; (**b**) antibacterial activity according to the type of oral biofilm; (**c**) antimicrobial resistance according to the type of oral biofilm; (**d**) antibacterial resistance according to the type of treatment and oral biofilm. Asterisks indicate statistical differences compared with the CHX and control (Ctrl) groups, respectively.

Figure 4 illustrates the antimicrobial activity of AgNPs against oral biofilms from individuals with and without MIDs, analyzed by sex. Generally, oral biofilms obtained from female and male participants showed similar MIC values (65.1 ± 84.2 and 58.7 ± 77.7 µg/mL, respectively), with no statistically significant differences (*p* = 0.139, Figure 4a). Although females had a tendency toward higher levels of antimicrobial resistance (49.9–101.6 µg/mL) in comparison with males (31.7–90.8 µg/mL) for specific oral biofilms, no statistical differences were identified (*p* > 0.05, Figure 4b). Moreover, the oral biofilms from patients with disabilities showed a tendency toward higher antimicrobial resistance (72.3 ± 100.5 µg/mL) than those from healthy patients (51.5 ± 54.7 µg/mL), with no significant differences (*p* > 0.05, Figure 4c,d). These results suggest that the antimicrobial effectiveness of the AgNPs was consistent among the different patient groups and genders and even significantly more effective than CHX in inhibiting the growth of oral bacteria. Also, the AgNPs exhibited the lowest microbial resistance across many of the oral biofilms.

### 2.5. Antimicrobial Substantivity of AgNP

Table 4 shows the general distribution of oral biofilm samples included in the antimicrobial substantivity assay. A total of 12 biofilm samples were analyzed, with uniform representation across the four study groups (three samples per group). The proportions of the biofilm samples were similar across all groups, showing no statistically significant variation (*p* > 0.05). In contrast, the ages differed among the study groups (*p* = 0.05), with the mean age increasing from the autism group (4.6 ± 0.0 years) and the no-disability group (4.3 ± 0.0 years) to the CP group (9.3 ± 0.1 years) and the DS group (12.6 ± 0.6 years). These results indicate that although the biofilm sample distribution was homogeneous, age, primarily among the DS subjects, differed across groups.

**Figure 5:**
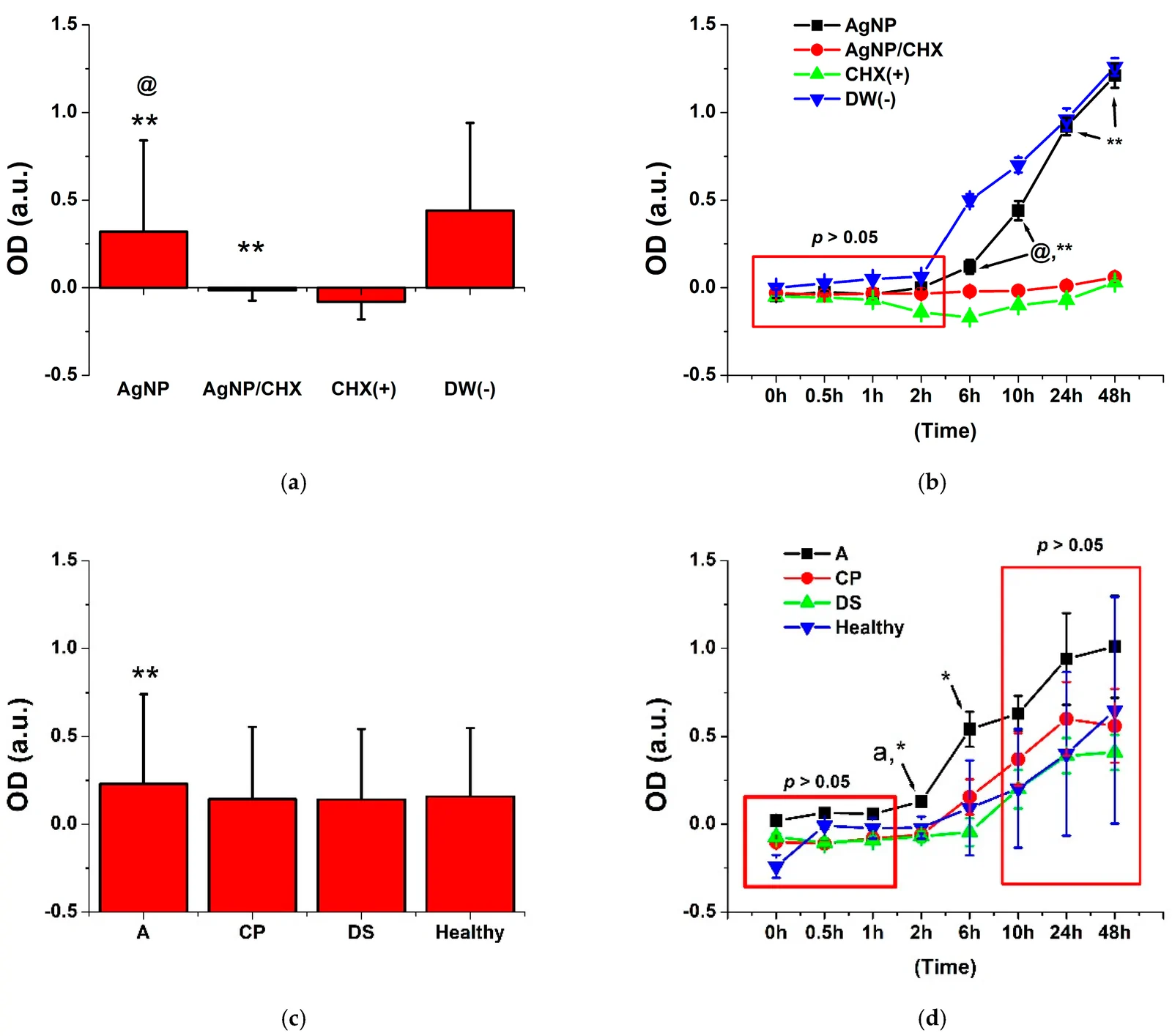
Antibacterial substantivity test of AgNPs in microbial biofilms from individuals with MIDs: (**a**,**b**) antibacterial substantivity according to the type of treatment; (**c**,**d**) antibacterial resistance according to the type of oral biofilm. Asterisks denote statistically significant differences relative to the negative control (DW) and oral biofilms from healthy individuals (* *p* < 0.05; ** *p* < 0.01). The symbol “@” denotes significant differences in comparison with the positive control (CHX) (*p* < 0.05). The letter “a” implies significant differences in comparison with the CP group (*p* < 0.05). Red squares denote comparisons without statistically significant differences (*p* > 0.05).

The antibacterial substantivity assay of AgNPs in oral biofilms from individuals with MIDs is presented in Figure 5. Treatment with AgNPs resulted in an adequate inhibition of bacterial growth (0.32 ± 0.52 a.u.) in comparison with the DW (0.44 ± 0.50 a.u.) (*p* < 0.05). In contrast, the combined treatment AgNP/CHX produced a better inhibitory effect on bacterial cell viability (−0.014 ± 0.06 a.u.) compared with AgNPs alone (0.32 ± 0.52 a.u.) (*p* < 0.05). In general, all treatments had significant antimicrobial variations over time (*p* < 0.001); however, specific times have been shown to have significant antimicrobial activity. At 6 and 10 h, AgNPs had better antimicrobial activity (0.12–0.44 a.u.) in comparison with DW (0.5–0.7 a.u.) (*p* < 0.05). Moreover, CHX and CHX combined with AgNPs exhibited the best antimicrobial activity against all biofilms at all time points (*p* < 0.05; Figure 5a,b). Additionally, the resistance profile of oral biofilms against antimicrobial agents was evaluated. Individuals with A showed the highest antimicrobial resistance levels (Figure 5c), with greater accelerated bacterial growth rates at 2 (0.12 ± 0.0 a.u.) and 6 h (0.54 ± 0.1 a.u.) compared with healthy patients (−0.02 ± 0.06 and 0.09 ± 0.27 a.u., respectively) (*p* < 0.05, Figure 5d). These results demonstrate that AgNPs significantly reduced bacterial proliferation compared with DW, while the AgNP/CHX combination and CHX alone showed the greatest inhibitory effects, supporting potential synergy or enhanced antimicrobial activity when AgNPs are combined with CHX. Additionally, specific times and types of oral biofilms were statistically involved in the antimicrobial activity of AgNPs.

**Figure 6:**
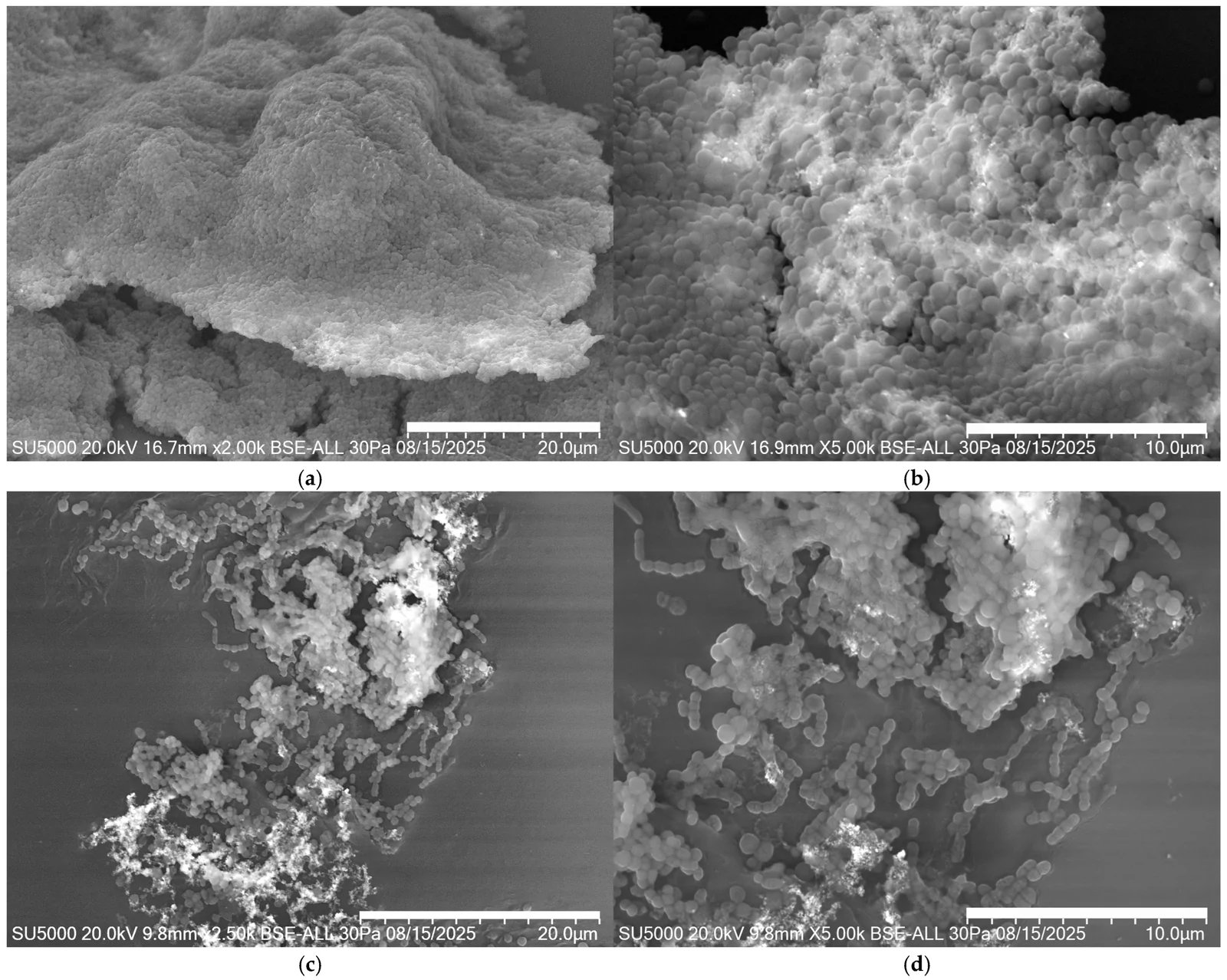
Micrographs of a representative biofilm from a healthy individual exposed to AgNPs: (**a**) oral biofilm not exposed, 2000×; (**b**) oral biofilm not exposed, 5000×; (**c**) oral biofilm exposed to AgNPs, 2500×; (**d**) oral biofilm exposed to AgNPs, 5000×. Images were generated with an Hitachi SU5000 FE-SEM instrument (Hitachi High-Tech Corporation, Tokyo, Japan) operated with an EM Wizard interface and EMIP-3 software (Electron Microscope Image Processing, Hitachi High-Tech Corporation, Tokyo, Japan), version 1.10.

FE-gSEM images from a representative oral biofilm exposed to AgNPs are illustrated in Figure 6. As shown in Figure 6a,b, untreated samples exhibited densely aggregated and firmly attached bacterial cells, forming a well-organized oral biofilm with strong cell–substrate and cell–cell interactions. The biofilm structure exhibited an estimated thickness ranging from 3 to 15 μm (Figure 6b). In contrast, bacterial cells exposed to AgNPs exhibited clear inhibition zones and disrupted biofilm architecture, characterized by altered adhesion patterns that weakened both intercellular interactions and cell–surface attachment (Figure 6c,d). Additionally, large and evident gaps with a low number of bacterial cells were identified in the biofilm structure exposed to AgNPs (Figure 6d).

## 3. Discussion

This study found that the AgNPs exhibited strong antimicrobial activity against dental biofilm collected from patients with MIDs. Overall, the AgNPs demonstrated excellent antibacterial activity, even outperforming CHX across all biofilm samples from both disabled and non-disabled patients. Specifically, biofilms from patients with autism exhibited significantly higher resistance to all antimicrobial solutions, including AgNPs, whereas biofilms from patients with PMR, NS, CPR, and CP showed the highest antimicrobial sensitivity (Figure 4b). Although no significant differences were observed according to gender, women showed a tendency toward higher antimicrobial resistance than men across all conditions. Although the microorganisms found in the biofilms from patients with MIDs exhibited greater initial bacterial growth than those from patients without disabilities, the bacterial inhibition of the AgNPs remained effective even in oral samples with increased bacterial growth. On the other hand, the antimicrobial substantivity of the AgNPs exhibited a notable decrease in bacterial growth in comparison with DW. Although the specific times of the AgNPs were shown to have greater antimicrobial effectiveness than the control group, the combination of the AgNPs with CHX and CHX alone had the highest inhibitory effects across all oral biofilms. Finally, antibacterial variations were assessed across oral biofilms, with subjects with autism showing the greatest sensitivity to all treatments. Thus, it may be suggested that the antimicrobial behavior of AgNPs could be related to their intrinsic physical and chemical properties and to specific microbiological characteristics associated with exposure time and type of biofilm, as well as the medical, dental, and demographic characteristics of each patient. To our knowledge, this is a pioneer study that evaluated the antimicrobial effects of AgNPs exposed to a wide spectrum of oral biofilms from participants with and without motor and intellectual incapacities, using a more robust sample size.

A variety of examinations have estimated the antibacterial performance of AgNPs exposed to multiple microbial species, highlighting the role of their physicochemical properties in determining their efficacy [37,42,43]. Authors have reported that the bactericidal and bacteriostatic activity of AgNPs can be modulated by physicochemical aspects, such as stability, particle dispersion, size, and other structural characteristics [44,45], while other authors have reported similar preparation methods for AgNPs [45,46,47]. In this investigation, the formation of AgNPs used silver nitrate as the starting precursor and gallic acid as the reducing agent, yielding sizes and shapes very similar to those previously reported in the literature [41,45,46,47]. Additionally, other reducing agents have been explored for the preparation of AgNPs, such as those obtained through organic methods using rosemary leaf extracts in volumes of 5–50 mL, while keeping the silver nitrate constant, yielding results very similar to those obtained when gallic acid is used as a reducing agent [48]. Studies have shown that pH is a very important factor in particle stability, helping prevent agglomeration. The affinity of the elements in gallic acid likely interferes with oxidation when they interact with phenolic groups, thereby reducing silver ions, enhancing stability, and preventing nanoparticle aggregation [49,50,51]. According to our results, an alkaline environment was established during nanoparticle synthesis by adjusting the pH to 11 using sodium hydroxide, yielding spherical particles with a size of 9.8 nm, which is consistent with previously reported findings [22] and favors antimicrobial activity. Also, the antimicrobial effectiveness of nanoparticles is strongly influenced by the zeta potential due to its impact on their ability to remain dispersed. Values within the range of −30 mV to +30 mV are typically associated with enhanced dispersion properties and reduced particle agglomeration [52,53]. In this study, the AgNPs exhibited appropriate electrical charges (−39.8 ± 3.3 mV), indicating stable nanoparticles and supporting their antimicrobial activity. In addition, the elemental composition of the AgNPs was not examined in this study, limiting an appropriate understanding of the characterization of the particles; however, recent reports, using a similar preparation of this nanomaterial, have already defined it [41]. Those reports have described a consistent Ag distribution in the total weight of samples (43.4 to 31.8%) [41]. It is probable that the amount of Ag present in the AgNPs used in this study is likely to have had a similar distribution in comparison with those reported works; therefore, an additional EDS analysis will be essential to confirm the presence and distribution of the chemical composition of the AgNPs.

The antibacterial impact of AgNPs has been widely examined against various microorganisms, including oral bacteria and biofilms [7,22,54,55,56]. Several studies have focused on evaluating individual microbial strains (planktonic bacteria) to determine the antimicrobial effect of AgNPs, using different sizes and types of bacteria [46,57,58]; however, few studies have analyzed the antimicrobial effect of AgNPs using clinical biofilms from patients with a particular condition. A study analyzed the antifungal effect of AgNPs in samples isolated from individuals with stomatitis, which indicated that AgNPs had antifungal activity, causing surface damage, cell alteration, and an antibiofilm effect, acting as a high-potential therapeutic for oral candidiasis [59]. Other studies have used similar sizes and shapes of AgNPs under similar chemical synthesis against bacterial isolates from participants with dental caries [39], periodontal disorder [40], and various endodontic conditions [41], demonstrating that AgNPs promote the inhibition of bacterial growth in microorganisms present within biofilms associated with the most prevalent oral infections. These authors conclude that AgNPs might serve as complementary antimicrobial therapies for controlling oral bacteria, with high potential for applications in dentistry [39,40,41]. Also, authors have evaluated the antimicrobial action of AgNPs deposited on titanium discs exposed to multispecies biofilms related to peri-implantitis (*Staphylococcus aureus* or various mixed bacteria), concluding that AgNPs developed excellent antimicrobial activity for all microbial samples, representing a promising therapy for the prevention of peri-implantitis [60]. In contrast, very few examinations have calculated the antibacterial behavior of AgNPs exposed to oral biofilms from individuals with MIDs. One investigation assessed the antimicrobial efficacy of size-dependent AgNPs in 37 oral biofilm samples collected from subjects with MIDs (*n* = 17) and individuals without MIDs (*n* = 20), including cases of Down syndrome, intellectual disability, and cerebral palsy [22]. Those authors concluded that AgNPs showed great antimicrobial action in all oral biofilms, relating the size and the characteristic of biofilms as the significant variables responsible for the antimicrobial effectiveness of the AgNPs, being a promising alternative for the control of the most representative bacteria found in oral diseases related to patients with MIDs [22]. Our results revealed that AgNPs exhibit excellent growth-inhibitory effects in all bacterial biofilms from individuals with MIDs. The results from antibacterial assays demonstrated a strong antimicrobial activity of AgNPs (46.3 ± 54.6 µg/mL) compared with CHX (84.4 ± 104.7 µg/mL) for any type of oral biofilm (*p* < 0.05). Specifically, patients with A and DS had significantly higher microbial resistance in comparison with patients with no disability (95.6 ± 113.9, 68.0 ± 122.6, and 51.5 ± 54.7 µg/mL, respectively), finding a higher frequency of sensitive biofilms to AgNPs than CHX. Likewise, microorganisms from patients with CP and NS tended to be the most sensitive (45.2–44.6 µg/mL), whereas bacteria from PMR and A subjects tended to be more resistant (100.4–95.6 µg/mL). In general, patients with disabilities were more resistant to all antimicrobials (72.3 ± 100.5 µg/mL) than subjects without disabilities (51.5 ± 54.7 µg/mL); therefore, no significant evidence was determined (*p* > 0.05). According to gender, no statistically significant differences were detected; however, women tended to have greater resistance to the antimicrobial effect of AgNPs (49.9–101.6 μg/mL) in comparison with men (31.7–90.8 μg/mL). These findings indicate that AgNPs provide effective bacterial growth inhibition against various types of oral biofilms from participants with and without MIDs, regardless of age or sex. Moreover, AgNPs demonstrated superior antimicrobial performance compared with CHX and showed lower microbial resistance in most biofilm types. AgNPs are known to possess distinctive characteristics associated with their extremely small size, stable surface properties, and efficient particle dispersion [61], with a high surface area, which promotes large surface–volume relationships, particularly with bacterial cells, facilitating antimicrobial susceptibility [37,62].

The mechanism of action of these metallic nanoparticles is still vague; however, authors have proposed that the antimicrobial effect of AgNPs is caused by various catalytic processes that occur during exposure of bacterial cells to free silver ions [61]. These physical and chemical interactions with the cell membrane of microorganisms, in which chemical affinities with thiol, amino, and hydroxyl components are involved, facilitate the penetration of particles or silver ions into the bacterial cell [36,63]. Inside, these silver components promote reactive oxygen species (ROS), which produce enzymatic deactivations, inhibition of DNA replication, denaturation of disulfide bonds from bacterial proteins, alteration of bacterial cell membranes, and lead to cell death [64]. Authors have proposed that Ag+ ions affect DNA replication, and, consequently, alter basic cellular functions. At the same time, Ag+ ions might interact with specific metabolic enzymes involved in glycolysis and energy production for bacterial functions, thereby affecting cell survival [61,65]. This study suggests that a complex and multifactorial mechanism of AgNPs occurs in oral biofilms due to the physicochemical properties of AgNPs but also the variability among oral biofilms, multispecies composition of biofilms, and microbiological variation in bacteria (as well as the clinical, sociodemographic, and personal characteristics of patients), which are simultaneously related to the antimicrobial behavior of these metallic nanomaterials.

Authors have also studied the combination of metallic nanoparticles with various agents, including antibiotics, to explore the synergic antimicrobial effect. The application of AgNPs in combination with blue light, antibacterial substances such as amoxicillin, or other metal-based nanoparticles (Fe, TiO_2_, Ni, ZnO, Au, and Cu, among others) has been investigated to improve their antibacterial effects, even against resistant microorganisms [66,67,68,69]. Our results from the substantivity antimicrobial assay indicate that AgNPs showed greater antimicrobial substantivity (0.32 ± 0.52 a.u.) than DW (0.44 ± 0.50 a.u.); however, the combination of AgNPs with CHX exhibited greater growth-inhibitory effects than AgNPs alone (*p* < 0.05). The exposure time acted as a relevant factor in the antimicrobial effect of the AgNPs. The growth-inhibition action of the AgNPs was significantly greater at 6 and 10 h (0.12–0.44 a.u.) in comparison with the DW group (0.5–0.7 a.u.), while CHX alone and CHX combined with AgNPs were the most effective at all time points and in the biofilm (*p* < 0.05). Once again, microorganisms in oral biofilms, particularly those from A subjects, showed the most resistance activity (0.12–0.44 a.u.) compared with biofilms from healthy patients (0.09 ± 0.27 a.u.) (*p* < 0.05); but, generally, all biofilms had similar levels of antimicrobial resistance (*p* > 0.05) (Figure 5c). At specific time points (2 and 6 h), biofilms from subjects with autism showed statistically higher levels of resistance than those from CP, DS, and healthy subjects (Figure 5d). In some cases, a tendency of biofilms from healthy subjects was identified to exhibit more resistance than those from DS and CP individuals (Table 3 and Figure 3c), but this difference was not statistically significant (*p* > 0.05). These discoveries suggest that AgNPs can limit and intervene in the formation of oral biofilms in patients with or without disabilities, creating an important synergistic effect when CHX is present. Although the mechanism of action of AgNPs might be influenced by various physical and chemical conditions, the type of oral biofilm and the exposure time were statistically associated with the bacterial growth inhibitory activity of AgNPs. It is known that many patients with disabilities are under medical treatment with various drugs, including antibiotics. Multiple variables, such as habits, oral hygiene, systemic and oral diseases, genetic factors, race, and microbial characteristics, among others, may influence this observed trend in the antimicrobial resistance [5,7,18].

Authors have not defined the antibiofilm mechanism of AgNPs against oral bacteria due to its complexity; however, some mechanisms have been suggested. Studies have shown that metal nanoparticles, such as AgNPs, can interact directly with EPS from mature or incipient biofilms during formation [70]. Most of these nanoparticles carry positive electrical charges on their surface; in contrast, molecules involved in EPS are negatively charged, resulting in electrostatic attraction between metallic particles and the biofilm. These physical constraints facilitate the disruption and alteration of metabolic processes in microorganisms, thereby interfering with the proper integration of the biofilm matrix [68,70]. This study supports the idea that the physicochemical characteristics of AgNPs are directly involved in the antibiofilm mechanism, but factors such as the type and microbiological condition of the biofilm, as well as dental, systemic, personal, sociodemographic, and other personal characteristics, are involved in the resistance or sensitivity of biofilm exposed to AgNPs. It is important to underline that the final concentration in the substantivity assay for the AgNPs was 5.1941 μg/mL, while the CHX solution used was 97.08 μg/mL; nevertheless, AgNPs, using a lower concentration in comparison with the CHX, could offer adequate and sustainable antimicrobial activity against any category of microbial biofilm. The antimicrobial substantivity of the AgNPs could be influenced by a prolonged absorption of AgNPs and the release of silver ions inside bacterial cells, limiting bacterial proliferation and the reorganization of microorganisms in a structured biofilm for a longer time [33]. It is probable that small particles released free silver ions and, electrically and chemically, interacted with the negative charges on bacterial membranes simultaneously with thiol, amino, and hydroxyl groups, facilitating the binding and penetration of these nanomaterials into bacterial cells [37,61]. Outside and inside the cell, Ag+ ions and small particles probably created and generated stress and ROS, interfering with vital cellular functions, including mitochondrial and ribosomal dysfunction, leading to reductions in ATP, enzyme inactivation, and protein denaturation, affecting the production and structure of the EPS matrix and, consequently, the adequate conformation of oral biofilm [71]. In parallel to this mechanism, a strong effect on “*quorum sensing*” must be observed, in which AgNPs produce a chemical discoordination among different species involved in the biofilm, affecting bacterial growth and the final configuration of the structure of the biofilm, leading to the disruption of the matrix [12,72].

On the other hand, the sociodemographic, clinical, and systemic conditions of the patients might also be related to antimicrobial sensitivity or resistance. Gender has been associated with antibiotic resistance in various studies and shows an important function in the mechanisms of antimicrobial behavior. Authors have reported that females have a higher bacterial growth rate and microbial resistance than men, permitting the presence and severity of oral diseases related to biofilms [73]. High levels of gamma interferon (INF-γ) production associated with the presence of estrogen facilitate the initiation and progression of oral diseases linked to bacterial biofilms, such as periodontitis [74]. These associations between gender and oral biofilm formation are unclear due to multiple factors involved in the underlying mechanisms; however, there is a strong predisposition toward the promotion of certain oral infections related to biofilms [75,76]. Our results show a strong predisposition among female subjects towards higher bacterial growth rates (0.084 ± 0.004 a.u.) and higher bacterial resistance (49.9–101.6 µg/mL) to both antimicrobials compared with male subjects (0.081 ± 0.004 a.u and 31.7–90.8 µg/mL), respectively); therefore, no significant data were found (*p* > 0.05). It is possible that physiological factors specific to women, including hormonal changes during puberty, menstruation, menopause, postmenopause, and pregnancy, as well as the use of birth control devices, such as as contraceptives, together with metabolic and immunological variations [40], may affect the interaction with AgNPs and contribute to differences in susceptibility or resistance. Another factor relevant to the antimicrobial activity of AgNPs is systemic conditions, particularly the presence of specific types of disability.

AgNPs have consistently been considered an alternative antimicrobial therapy with high potential for biomedical and, particularly, dental applications; however, their safe use and non-toxic effects in animals and humans remain questionable. The increased use of products containing AgNPs has created various environmental and health problems stemming from a poor understanding of the toxicity mechanisms of these nanomaterials [77,78]. Authors have proposed that AgNPs or Ag+ ions can cross biological membranes and directly penetrate cells [77]; once internalized, these metallic nanoparticles can accumulate in various tissues and organs, such as the kidneys, liver, lungs, brain, stomach, intestines, heart, and spleen, potentially leading to physiological alterations [79,80,81]. Endogenous factors, such as the shape, size, surface, type, and other physicochemical characteristics, can promote relevant toxicological manifestations, while external factors, such as the dosage, administration route, sensitivity, exposure time, and various particularities and medical conditions of patients, can also be related to the toxicological effects of AgNPs [53,79,81,82,83]. Nevertheless, studies have revealed that the toxicity of AgNPs is not always significant in in vivo models and humans, reporting that the use of AgNPs was found to be systemically available [79], with no altered hematological and biochemical values [83] and no detectable toxicity [82], highlighting the importance of controlling factors such as administration routes, dosages, exposure time, concentration, and sensitivity systems, as well as the wide variations in the physicochemical characteristics of AgNPs [82,84,85]. It is very possible that the combination of AgNPs and CHX could result in a greater cytotoxic effect while, simultaneously, offering good antibiofilm activity. Undoubtedly, further studies are required to clarify the safe use of AgNPs and their potential combinations for the control of oral biofilms.

Although the results support the potential application of AgNPs as an alternative antimicrobial therapy for managing bacterial growth within biofilms from patients with motor and intellectual disabilities, it is very important to explore other antimicrobial, antibiofilm, and toxicity mechanisms based on different and more rigorous experimental methods using in vitro and in vivo designs, with more accurate and simulated conditions according to the oral cavity. Also, this work highlights that a SEM analysis was conducted using a single exploratory sample from a subject without a disability, limiting the generalization and extrapolation of the antibiofilm property of the AgNPs. Surely, new and novel methodological routes, including a significant sample size, different mental and intellectual conditions, systemic and dental characteristics, and different races (e.g., Asian and European), as well as different nanostructured materials, could offer a better understanding and increased efforts to control and prevent oral diseases related to bacterial biofilms that affect the life expectancy of individuals with mental and motor disabilities.

## 4. Materials and Methods

### 4.1. Preparation and Characterization of AgNPs

AgNPs were synthesized according to previously described methodologies (2024 and 2025) [22,41]. Briefly, a 0.01 M AgNO_3_ solution (silver nitrate, CTR Scientific, Monterrey, Mexico) was prepared by dissolving the compound in 100 mL of deionized water under magnetic stirring for 5 min in a 250 mL glass container. Subsequently, 10 mL of deionized water containing 0.1 g of C_7_H_6_O_5_ (gallic acid, Sigma-Aldrich, St. Louis, MI, USA) was incorporated into the silver nitrate solution, followed by an immediate adjustment of the pH to 11 using 1.0 M NaOH (sodium hydroxide, Jalmek Scientific, San Nicolás de los Garza, Mexico). The resulting mixture was maintained under continuous stirring for an additional 10 min at room temperature.

Particles were hydrodynamically and electrically characterized using dynamic light scattering (DLS) in a particle analyzer instrument (HORIBA Scientific, Nano Partica SZ-100, Piscataway, NJ, USA). Before DLS measurements, the solution was stirred to improve the dispersion of the particles and then diluted with deionized water (DW) to avoid multi-dispersion phenomena. The analysis was conducted with a detection angle of 90 °C, with at least three different independent measurements per sample at 25 °C. For electrical properties, solutions were diluted with DW and placed in specific capillary cells for zeta potential analysis. The evaluation was made at 25 °C in triplicate, and the results are expressed in millivolts (mV). Transmission electron microscopy (TEM) analysis was performed with a JEOL JEM 2200-FS-CS (2006 model, Tokyo, Japan), with an accelerating voltage of 200 kV. For this analysis, the AgNP solution was stirred using a magnetic stirring plate for 2 min to disperse the nanoparticles. After, 10 μL of this mixture was deposited onto a carbon-coated copper grid, remaining on the grid for 2 min to permit particle absorption. The grid was dried at room temperature under dust-free conditions until completely dry. The preparation and evaluation by DLS of each AgNP solution was always conducted together, at most, every three months to avoid changes in their properties.

### 4.2. Patient Recruitment

A cross-sectional consecutive study using non-probabilistic sampling was conducted at the Multidisciplinary Oral Care Clinic of the Department of Stomatology at the Autonomous University of Juarez City from January 2022 to January 2023. Written informed consent was acquired from all individuals before the collection of microbial biofilm samples. For individuals with MIDs, authorization was provided by their parents and/or legal guardians following the ethical standards summarized in the Declaration of Helsinki. The investigation protocol was checked and authorized by the Institutional Ethics Committee of the Institute of Biomedical Sciences, Autonomous University of Ciudad Juárez (ICB-UACJ), under control number CEI-2023-1-70. Fifty-four patients aged between 3 and 26 years decided voluntarily to participate in this study. The selection criteria were established according to the most prevalent disabilities presented by patients from the Multidisciplinary Clinic for oral care, as follows:

Inclusion criteria:

- Subjects of any age;
- Subjects of any gender;
- Subjects with any motor and intellectual disability diagnosed by a medical doctor.

Exclusion criteria:

- Subjects who underwent professional dental care or antibiotic therapy within the last two months;
- Subjects with relevant systemic diseases;
- Subjects with unclear or undefined medical diagnoses.

Participants were classified based on the presence and categories of MIDs, as follows: (a) patients with MIDs and (b) patients without MIDs (control group). The presence and type of MIDs were confirmed by the legal guardian of each patient.

### 4.3. Collection of Oral Biofilms

The collection of oral biofilms was conducted using the method previously reported by Holguin-Meraz et al. (2024) [22]. Using a sterilized wooden toothpick (10 cm in length and 2 cm in width) with a fat base and a pointed tip, mechanical sweeps in the gingival margin were performed to obtain oral biofilms. To standardize the biofilm sampling, teeth from the left mandibular region were selected, with a slight subgingival insertion. In cases where mandibular teeth were absent, maxillary teeth were used as an exception.

The collected oral biofilm samples were transferred into standard 10 mL test tubes containing Mueller-Hinton broth (MH, BD™ Difco™, Rockville, MD, USA) and stored under incubation at 37 °C for 24 h. The collection of oral biofilms was carried out by an expert in the dental field and patients with special needs, with the support of residents from various dental specialties.

### 4.4. Microbial Growth Rate and Standardization of Microbial Suspension

Microbial growth kinetics were evaluated after the first 24 h of incubation by mixing 3 mL of phosphate-buffered saline (PBS, pH 7.4) with 100 µL of each microbial suspension. Bacterial proliferation was quantified by measuring the optical density (OD) at 550 nm with an Eppendorf BioPhotometer Plus spectrophotometry instrument (Hamburg, Germany). All measurements were performed in triplicate. Subsequently, a standardized microbial suspension including 1.5 × 10^6^ colony-forming units per milliliter (CFU/mL) was set corresponding to the McFarland scale and used for all subsequent microbiological evaluations.

### 4.5. Antimicrobial Activity of AgNPs in Microbial Biofilms

The antimicrobial assessment was conducted following a reported protocol [22]. Briefly, oral samples were cultured in Mueller-Hinton broth (MH) and incubated at 37 °C for 24 h. The minimum inhibitory concentrations (MICs) of the AgNP solutions were subsequently determined using a 96-well microdilution assay with serial dilutions. Wells were inoculated with 100 µL of a standardized bacterial suspension containing approximately 1.5 × 10^6^ CFU/mL and incubated under the same conditions for an additional 24 h. The first and twelfth lines of the microplate were designated as negative and positive controls, respectively. MIC values were established based on the visual and stereomicroscopic evaluation of the bacterial turbidity. A chlorhexidine solution (CHX, Consepsis™, Ultradent Products Inc., South Jordan, UT, USA) with a concentration of 2.0% was included as the reference antimicrobial treatment under identical experimental conditions. All experiments were performed in triplicate.

In addition, a field-emission scanning electron microscopy instrument (FE-SEM, Hitachi, model SU5000, Hitachi High-Tech Corporation, Tokyo, Japan), with an accelerating voltage of 20 kV, was applied to explore the antimicrobial and antibiofilm behavior of nanoparticles in a single oral biofilm randomly collected from patients without disability. After the antibacterial assay, the wells containing bacterial cells exposed to AgNPs at their respective minimum inhibitory concentration (MIC), along with the negative control wells, were fixed using a glutaraldehyde solution with a concentration of 0.1% for 5 min. The samples were then rinsed three times with phosphate-buffered saline (PBS, pH 7.4) and dehydrated by sequential immersion in ethanol using increasing concentrations (50%, 60%, 70%, 90%, 95%, and 100%) for 20 min at 4 °C. After dehydration, the wells were allowed to air dry and were subsequently covered with a thin film of gold by sputter coating. Cell morphology, biofilm structure, and cell–cell and cell–surface interactions were evaluated.

### 4.6. Antimicrobial Substantivity of AgNPs on Oral Biofilms

The antimicrobial substantivity assessment was directed to measure the capacity of the AgNPs to retain the residual antimicrobial activity following application and to inhibit bacterial proliferation, as well as the development of more mature and organized biofilms. The antimicrobial substantivity was carried out according to previous reports [41,86] with modifications. Using convenience sampling, twelve oral biofilm samples (three samples per condition) were randomly selected based on the most relevant disabilities (A = autism, CP = cerebral palsy, DS = Down syndrome, and no disability). The type of disability was chosen according to the most frequent disabilities treated at the multidisciplinary oral health clinic. For the antimicrobial substantivity, 20 μL of each tested solution (AgNPs, chlorhexidine, and deionized water) was transferred into sterile tubes containing 4 mL of Mueller-Hinton broth. After, 100 μL of a standardized microbial suspension (1.3 × 10^8^ CFU/mL) was placed in each tube. In addition, the synergistic influence of the AgNPs combined with CHX was evaluated with a 1:1 ratio mixture under the same bacteriological conditions. Following incubation at 37 °C, microbial growth variations were monitored by analysing absorbance changes at 550 nm with a UV–Vis spectrophotometry instrument (Jenway™ 7305, Bibby Scientific™, Stone, Staffordshire, UK) at different moments (0, 0.5, 1, 2, 6, 10, 24, and 48 h). All tests were completed in triplicate, and the effects were reported as absorbance units (a.u.).

### 4.7. Statistical Analysis

The findings reported from the different microbiological assays were examined with descriptive and inferential statistics. The homogeneity among the study groups according to the MIDs was evaluated using a Pearson’s chi-square analysis. The Kolmogorov–Smirnov test was used to determine the normality of the antimicrobial findings. Kruskal–Wallis (for nonparametric variables) or ANOVA (parametric variables) analysis was used for multiple comparisons. Pairwise comparisons were applied to Dunn’s test with Bonferroni correction to control for the error associated with multiple comparisons. Independent comparisons of nonparametric and parametric values were reviewed with the Mann–Whitney U test and t-test, respectively. For antimicrobial substantivity, the Friedman test was applied to independent variables related to multiple points. Statistical examinations were conducted with IBM-SPSS software (version 25, SPSS, Chicago, CA, USA), and statistical significance was established at *p* < 0.05.

## 5. Conclusions

The antimicrobial effectiveness of AgNPs was confirmed in microbial oral biofilms collected from individuals with and without motor and intellectual incapacities. Antimicrobial resistance was found to depend on the type of biofilm with oral microorganisms from autistic and Down syndrome subjects (95.6 ± 113.9 and 68.0 ± 122.6 µg/mL, respectively) showing the most resistance. Nevertheless, AgNPs exhibited growth-inhibitory activity (46.3 ± 54.6 µg/mL), even against resistant biofilms with high bacterial growth rates (PMR = 0.22 ± 0.0 a.u.; A = 0.13 ± 0.0 a.u.; DS = 0.11 ± 0.0 a.u.; and CPR = 0.09 ± 0.0 a.u.; among others). The antimicrobial substantivity of AgNPs consistently reduced bacterial growth in common biofilms from disabled patients (0.32 ± 0.52 a.u.) at specific time points, using lower concentrations than CHX (AgNPs = 5.1941 μg/mL and CHX = 97.08 μg/mL); however, the mixture of AgNPs and CHX caused the strongest antimicrobial substantivity (*p* < 0.001). A multifactorial complex mechanism of AgNPs is likely driven by intrinsic particle properties and external patient conditions, determining the antimicrobial efficiency of these nanomaterials; however, this information should be further investigated. Although AgNPs have the potential to control and prevent oral diseases associated with bacterial biofilms in patients with and without MIDs, further innovative experimental investigations in the biomedical field on their efficiency, safe use, and long-term effects must be explored.

## Figures and Tables

**Table 1:** Physical characterization of the AgNPs.

AgNPs (nm)	DLS Diameter (nm)	Shape	Initial Concentration (μg/mL)	Zeta Potential (mV)
9.8 nm	9.8 ± 1.7	Spherical	1070	−39.8 ± 3.3

DLS = dynamic light scattering. The values obtained for the zeta potential and DLS are presented as the average and standard deviation (SD).

**Table 2:** Sociodemographic distribution of individuals with and without disabilities.

	Motor and Intellectual Disability (*n* = 26 Subjects)							No Disability (Controls) (*n* = 28 Subjects)	Total (*n* = 54 Subjects)	*p*-Value
	A (*n* = 9 Subjects)	CS (*n* = 2 Subjects)	CP (*n* = 6 Subjects)	DS (*n* = 6 Subjects)	PMR (*n* = 1 Subject)	NS (*n* = 1 Subject)	CPR (*n* = 1 Subject)			
	Mean ± standard deviation (years)									
Age	6.4 ± 0.5	7.0 ± 0.3	11.0 ± 1.1	12.1 ± 1.8	22	9	19	8.2 ± 0.3	9.1 ± 6.0	0.001 *
Male	5.2 ± 0.4	6.0 ± 0.0	14.2 ± 1.1	11.6 ± 2.8	-	-	-	9.5 ± 0.6	9.1 ± 6.0	0.984
Female	8.0 ± 6.8	8.0 ± 0.0	4.5 ± 0.5	12.6 ± 2.5	22	9	19	7.0 ± 0.2	9.1 ± 4.0	
	Frequency (%)									
Gender										
Male	5 (55.6)	1 (50)	4 (66.7)	3 (50)	1 (100)	1 (100)	1 (100)	14 (50)	30 (55.6)	0.068
Female	4 (44.4)	1 (50)	2 (33.3)	3 (50)	0 (0)	0 (0)	0 (0)	14 (50)	24 (44.4)	

A = autism; CS = craniosynostosis; CP = cerebral palsy; DS = Down syndrome; PMR = psychomotor retardation; NS = Noonan syndrome; CPR = congenital progeria. Statistical differences in age and sex were measured using a t-test and chi-square test, respectively. An asterisk (*) denotes a statistical difference (*p* < 0.05).

**Table 3:** Antimicrobial effectiveness of the AgNPs in the oral biofilms from participants with and without motor and intellectual incapacities.

	MIC (µg/mL)	*p*-Value
Antimicrobials		
AgNP	46.3 ± 54.6	<0.000
CHX	84.4 ± 104.7	
Gender		
Female	65.1 ± 84.2	0.139
Male	58.7 ± 77.7	
Oral biofilms		
A	95.6 ± 113.9	0.129
CS	64.0 ± 99.6	
CP	45.2 ± 40.5	
DS	68.0 ± 122.6	
PMR	100.4 ± 134.2	
NS	44.6 ± 31.3	
CPR	66.8 ± 81.7	
No disability (controls)	51.5 ± 54.7	

MIC results are expressed as the mean and standard deviation. A = autism; CS = craniosynostosis; CP = cerebral palsy; DS = Down syndrome; PMR = psychomotor retardation; NS = Noonan syndrome; CPR = congenital progeria. Kruskal–Wallis, t-Student, and U Mann–Whitney tests were employed to determine statistical differences among oral biofilms, gender, and antimicrobials, respectively (*p* < 0.05).

**Table 4:** Distribution of the oral biofilms used for the antimicrobial substantivity test.

	Oral Biofilms *n* = 12 Samples (%)	Age (Years) Mean ± SD
Disability		
A	3 (25)	4.6 ± 0.0
CP	3 (25)	9.3 ± 0.1
DS	3 (25)	12.6 ± 0.6
No disability	3 (25)	4.3 ± 0.0
Total	12 (100)	7.7 ± 6.0
*p*-Value	>0.05	0.05

A = autism; CP = cerebral palsy; DS = Down syndrome. Age is expressed as the mean and standard deviation. Chi-square and Kruskal–Wallis tests were applied to determine statistical variations among the distributions of oral biofilms and age, respectively.

## Data Availability

The original contributions presented in this study are included in the article. Further inquiries can be directed to the corresponding author.
